# Simultaneous finding of chronic lymphocytic leukemia and residual hairy cell leukemia using a lymphocyte‐binding anti‐CD antibody microarray

**DOI:** 10.1002/ccr3.1416

**Published:** 2018-02-13

**Authors:** Alina N. Khvastunova, Liubov S. Al‐Radi, Olga S. Fedyanina, Sofya A. Kuznetsova

**Affiliations:** ^1^ Dmitry Rogachev National Research Centre of Pediatric Hematology Oncology and Immunology, Russian Ministry of Health 1 Samory Mashela st. Moscow 117997 Russia; ^2^ Centre for Theoretical Problems of Physicochemical Pharmacology RAS 4 Kosygina st. Moscow 119991 Russia; ^3^ Research Center for Hematology Russian Ministry of Health 4 Novy Zykovsky proezd Moscow 125167 Russia

**Keywords:** Antibody microarray, chronic lymphocytic leukemia, cluster of differentiation, hairy cell leukemia, morphology

## Abstract

The morphologic diagnosis of hairy cell leukemia coexisting with another lymphoproliferative disorder is hindered by the small size of hairy cell population. It can be simplified by presorting peripheral blood mononuclear cell using an anti‐CD antibody microarray on transparent support (including anti‐CD11c, CD25, CD103, and CD123) before their morphology analysis.

The morphologic diagnosis of hairy cell leukemia (HCL) is not easy, as the patients are usually leukopenic and hairy cells sometimes constitute not more than several percent of total peripheral blood leukocytes. The diagnosis can be even more challenging if HCL coexists with another lymphoproliferative disorder such as B‐cell chronic lymphocytic leukemia (CLL) especially when HCL is not a predominant component. Here we show a clear separation of two leukemic cell populations in a patient with concomitant HCL and CLL using peripheral blood mononuclear cells (PBMC) presorting on an anti‐CD antibody microarray therapy.

The patient, an 80‐year‐old man, has been treated for hairy cell leukemia at the age of 67 and has achieved complete remission after α‐interferon and cladribine treatment at that time. After more than 10 years, he presented with low platelet counts (WBC 7.8 × 10^9^/L, PLT 57 × 10^9^/L), high lymphocyte percentage (76%), moderate peripheral adenopathy, and splenomegaly 133 × 62 mm and was suspected to have a relapse on HCL. Morphology and cytoplasmic tartrate‐resistant acid phosphatase (TRAP) activity of his peripheral blood mononuclear cells (PBMC) were analyzed after presorting by their surface cluster of differentiation (CD) antigens using an anti‐CD antibody microarray on a transparent support 1 for preliminary diagnosis (Fig. 1A–C). Eighty six percent of patient's lymphocytes were found to have mature small lymphocyte morphology with occasional prolymphocytes (Fig. 1D) and to express surface CD5, CD19, CD20, CD22, CD23, CD25, CD27, CD43, CD200, and *κ* Ig light chains. About 0.1% of patient's lymphocytes were hairy cells (Fig. 1E) expressing CD11c, CD19, CD20, CD25, CD103, CD123. About half of the hairy lymphocytes (0.05% of total lymphocyte count) had strong TRAP activity (Fig. 1F). These findings suggested the coexistence of chronic lymphocytic leukemia (CLL) with minimal residual hairy cell leukemia (HCL) in accordance with ESMO clinical practice guidelines for HCL and CLL diagnosis 2, 3. PBMC flow cytometry detected a monoclonal B‐cell population (more than 80% of total B‐cell content) with CD19+/*κ*+/CD5+/CD23+/CD20+/CD43+/CD38− immunophenotype typical for CLL 3 and a small lymphocyte population (0.013% of total peripheral blood leukocytes) with CD19+/CD11c+/CD103+/LAIR‐1+/CD25+ immunophenotype typical for HCL 2. The patient was diagnosed with chronic lymphocytic leukemia (Binet A, Rai 0) with minimal residual hairy cell leukemia and required no treatment. At present (2 years after being first diagnosed with CLL), he is still under observation, has CLL and residual HCL with WBC 14.8 × 10^9^/L, PLT 54 × 10^9^/L, lymphocytes 75%, and requires no treatment.

**Figure 1:**
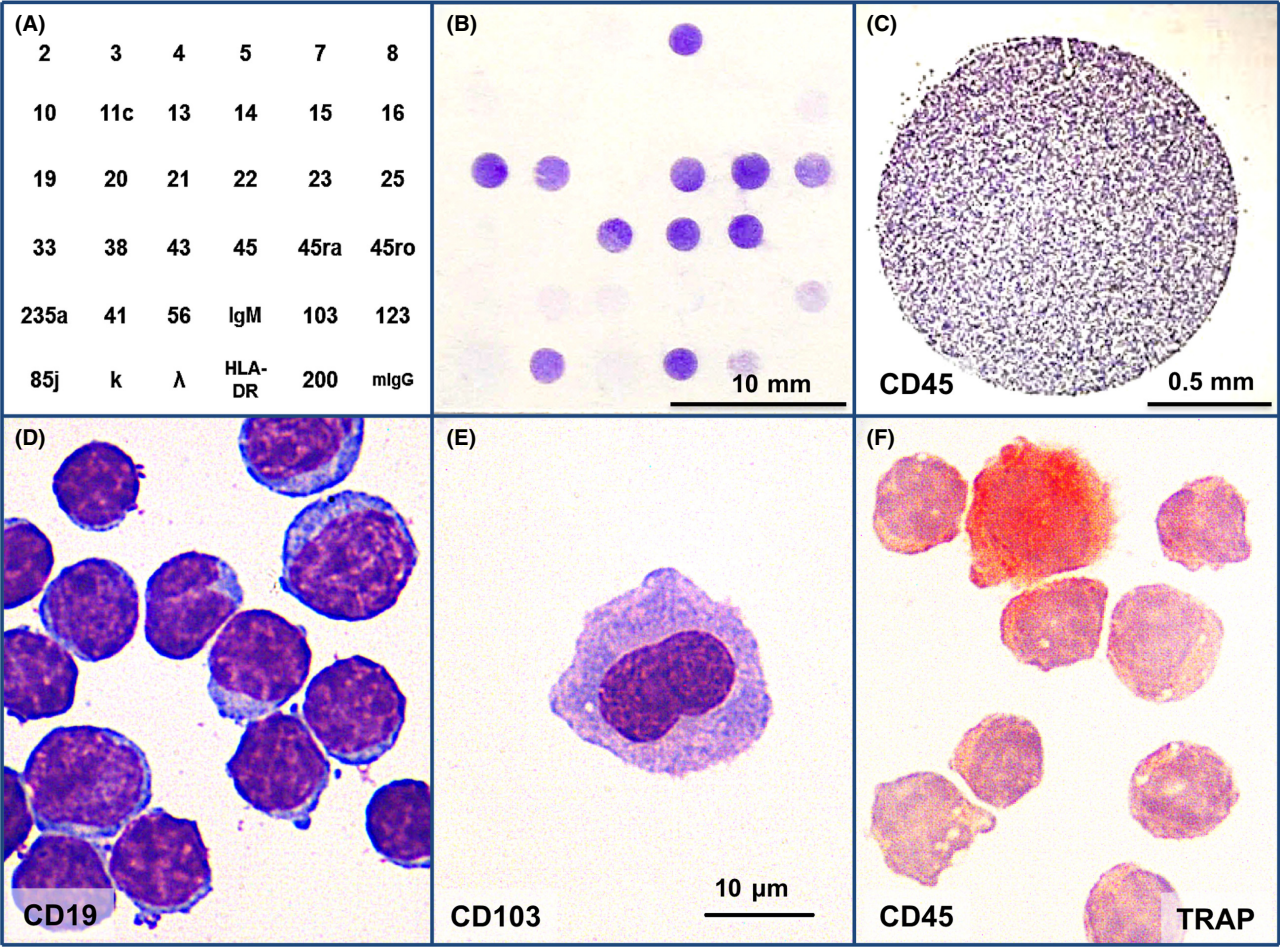
The patient's peripheral blood mononuclear cells bound to the anti‐CD antibody microarray. (A) The microarray map, the numbers indicate the position of spotted antibodies against corresponding CD antigens; *κ* and *λ* correspond to anti‐*κ* and anti‐*λ* Ig light‐chain antibodies, mIgG is a mixture of mouse IgG (negative control); (B‐E) The microarray with bound patient's PBMC after May‐Grunwald‐Giemsa staining: the cells bound to anti‐CD45 antibody (C), to anti‐CD19, x1000 (D), to anti‐CD103, x1000 (E); (F) the tartrate‐resistant acid phosphatase reaction in anti‐CD45‐bound patient's PBMC demonstrated by pararosanilin method, x1000.

Both synchronous and metachronous existence of HCL and CLL are extremely rare but have been described before as well as HCL developing among CLL patients and CLL developing among HCL patients 4, 5. The exact reasons for such coexistence are not known, and the immunoglobulin gene rearrangement studies seem to indicate the common origin in some of reported cases and different clonal origins in the others 5. Here we show that anti‐CD antibody microarray permits to observe circulating HCL cells in concentrations as low as 0.1% from total PBMC content due to the presence of antibodies against CD103 and CD123 in its panel, as CD103 and CD123 are expressed on hairy cells but absent on virtually all normal PBMCs. This high sensitivity makes the microarray suitable for diagnosis of hairy cell leukemia coexistence with other lymphoproliferative disorders as well as for detection of minimal residual disease in HCL with sensitivity compatible with the flow cytometry criteria of hairy cells constituting >0.3% of peripheral blood leukocytes 6.

## Authorship

AK: performed the microarray analysis and wrote the paper, LAR: was involved in clinical care of the patient and edited the paper, OF: performed the microscopy, SK: wrote the paper.

## Conflict of Interest

None declared.
